# Discriminating lymphomas and reactive lymphadenopathy in lymph node biopsies by gene expression profiling

**DOI:** 10.1186/1755-8794-4-27

**Published:** 2011-03-31

**Authors:** To Ha Loi, Anna Campain, Adam Bryant, Tim J Molloy, Mark Lutherborrow, Jennifer Turner, Yee Hwa Jean Yang, David DF Ma

**Affiliations:** 1 Blood Stem Cell and Cancer Research Unit, Department of Haematology, St Vincent's Hospital, Victoria Street, Darlinghurst, Australia; 2 Centre for Mathematical Biology, School of Mathematics and Statistics, University of Sydney, Sydney, Australia; 3 Department of Anatomical Pathology, St Vincent's Hospital, Victoria Street, Darlinghurst, Australia

## Abstract

**Background:**

Diagnostic accuracy of lymphoma, a heterogeneous cancer, is essential for patient management. Several ancillary tests including immunophenotyping, and sometimes cytogenetics and PCR are required to aid histological diagnosis. In this proof of principle study, gene expression microarray was evaluated as a single platform test in the differential diagnosis of common lymphoma subtypes and reactive lymphadenopathy (RL) in lymph node biopsies.

**Methods:**

116 lymph node biopsies diagnosed as RL, classical Hodgkin lymphoma (cHL), diffuse large B cell lymphoma (DLBCL) or follicular lymphoma (FL) were assayed by mRNA microarray. Three supervised classification strategies (global multi-class, local binary-class and global binary-class classifications) using diagonal linear discriminant analysis was performed on training sets of array data and the classification error rates calculated by leave one out cross-validation. The independent error rate was then evaluated by testing the identified gene classifiers on an independent (test) set of array data.

**Results:**

The binary classifications provided prediction accuracies, between a subtype of interest and the remaining samples, of 88.5%, 82.8%, 82.8% and 80.0% for FL, cHL, DLBCL, and RL respectively. Identified gene classifiers include LIM domain only-2 (*LMO2*), Chemokine (C-C motif) ligand 22 (*CCL22*) and Cyclin-dependent kinase inhibitor-3 (*CDK3*) specifically for FL, cHL and DLBCL subtypes respectively.

**Conclusions:**

This study highlights the ability of gene expression profiling to distinguish lymphoma from reactive conditions and classify the major subtypes of lymphoma in a diagnostic setting. A cost-effective single platform "mini-chip" assay could, in principle, be developed to aid the quick diagnosis of lymph node biopsies with the potential to incorporate other pathological entities into such an assay.

## Background

The increasing complexity of lymphoma diagnosis and classification is the result of a vastly improved understanding of its underlying molecular pathogenesis and resultant attempts to group subtypes of lymphoma in a clinically and biologically meaningful manner. From a practical perspective, this increasing complexity places great demands on the pathologist. Accurate diagnosis and classification of lymphoma in lymph node biopsies requires pathological evaluation utilising morphological analysis of an acceptable biopsy specimen, together with a series of supplementary tests including immunophenotyping by immunohistochemistry and flow cytometry, and increasingly cytogenetics, FISH and PCR data [1]. Such ancillary tests can be costly and time consuming, requiring specialised technicians and analytical experience from multiple divisions of a pathology laboratory. Of benefit would be a cost-effective, single platform ancillary test that provides a rapid standardised diagnosis of lymphoma and recognition of major subtypes, allowing more selective use of other ancillary tests during subsequent assessment by the pathologist.

Genome-wide gene expression profiling (GEP) is a novel approach to disease classification based on the molecular biology of the disease. This 'genetic fingerprint' data thus allows the identification and classification of individual tissue samples according to their distinct gene expression profiles. There is a significant body of research employing GEP in lymphoma, having been used for a number of purposes including distinguishing closely related lymphoma phenotypes such as diffuse large B-Cell lymphoma (DLBCL) versus Burkitt lymphoma [2] and DLBCL from primary mediastinal B-cell lymphoma [3], to trace malignant lymphoma phenotypes to "normal cell of origin" [4] and to identify expression profiles linked to tumour prognosis [5]. However, there is a lack of publications that address the potential value of gene expression microarray in aiding the routine diagnosis and classification of lymphoma in tissue biopsies from individual cases suspected of lymphoma. The feasibility of GEP as a tool to classify tumour tissue has been examined for other cancers, such as breast [6], colon [7], prostate [8] and renal tumours [9].

Distinct from previous microarray studies of lymphoma, this single institute study evaluated whether gene expression microarray as a single platform could be used to distinguish three major subtypes of lymphoma and non-malignant reactive lymphadenopathy (RL) in individual lymph node samples. To our knowledge, this study is a first attempt to apply such a strategy to lymph node specimens across different subtype diagnoses in a diagnostic setting. Our heterogeneous study set enabled the identification of gene signatures that are likely an accurate representation of each diagnostic type, given that this was determined by comparing each diagnostic type against the remaining cases by binary classification approach.

## Methods

### Patient samples

Lymph node specimens from patients undergoing biopsy for suspected lymphoma were identified by the Department of Pathology at St Vincent's Hospital, Sydney. Samples were collected subject to written consent for this human research ethics approved study (H00/028/1). A portion of the fresh biopsy specimen (≥2 mm^3^) was collected in RNAlater solution (Ambion, Foster City, CA) and then cryopreserved in liquid nitrogen. A further five biopsy samples (two cHL, two DLBCL, one FL) were obtained from the Newcastle Mater Misericordiae Hospital. The diagnosis and classification of each specimen was made or reviewed by an expert haematopathologist (JT) practising at our centre [10], according to the WHO Classification of Tumours of Haematopoietic and Lymphoid tissue 2001 [11]. All samples were collected during 2001-07 and analysed prior to the 2008 update of this classification system. Most cases (*n *= 16) of DLBCL were subclassified into germinal centre B (GCB) and non-GCB cell types (*n *= 5 and *n=*11 respectively) by immunohistochemistry according to the Hans algorithm [12]. The 23 cases of RL included reactive hyperplasia (*n *= 16), reactive hyperplasia with progressive germinal centre transformation (*n *= 3) or granuloma related to toxoplasmosis (*n *= 1), dermatopathic lymphadenitis (*n *= 1) and normal (*n *= 2).

### RNA and Microarray assays

Frozen lymph node tissue was homogenised in TRIzol reagent (Invitrogen, Victoria, Australia) using a pellet pestle and total RNA isolated using RNeasy micro-column purification (Qiagen, Doncaster, Australia). The integrity of total RNA was assessed by denaturing agarose gel electrophoresis (1% agarose, 221 mM formaldehyde, 20 mM MOPS, 5 mM sodium acetate, 1 mM EDTA). Only samples with distinct 28 S and 18 S RNA bands were assayed by microarray. The total RNA extracted from granulocyte colony-stimulating factor mobilised peripheral blood stem cells (PBSC) samples from 10 healthy individuals (collected with informed consent) were pooled and used as the reference RNA for microarray assays. Two-colour fluorescent probe synthesis was carried out on 2 μg of patient (Cy5 - Red) and reference (Cy3 - Green) RNA and then competitively hybridised to a microarray using the 3DNA 900MPX kit (Genisphere, Hatfield, PA) according to the manufacturer's protocol. A GenePix 4000A scanner and GenePix Pro 3.0 image analysis software (Molecular devices, Sunnyvale, CA) was used to capture microarray images and quantify fluorescent signals from each feature. The microarrays used in this study were printed by the Adelaide Microarray Facility (Adelaide, Australia) using the Compugen library of 19000 70-mer oligonucleotides, which covers over 12000 generic human genes.

### Preprocessing of array data

Data from GenePix result files were pre-processed by within-array print-tip Lowess normalisation. The quality of each array was assessed prior to analysis to ensure only arrays of sufficient quality were retained. A quality score was obtained using the QC CV scoring from arrayQuality [13]. The microarray data with clinical information have been deposited in NCBI's Gene Expression Omnibus (GEO, http://www.ncbi.nlm.nih.gov/geo/) and are accessible through GEO Series accession number GSE23647. This study is comprised from two batches of arrays developed over two years. The batches were analysed and normalised separately to maintain the independence of the two datasets.

### GEP classification analysis

The statistical analysis was performed using the R statistical software version 2.8.1. Microarray gene expression data from 81 patients of batch-1 arrays (training set) were used to develop a diagnostic profile. An independent series consisting of a further 35 patients (batch-2 arrays - test set) were used to independently assess the classification accuracy of the profile. The patient information, diagnosis and number of samples used in the training and test dataset of this study are indicated in Table 1.

**Table 1:** Summary of the biopsies in each disease category examined by microarray

	Training set (*n *= 81)	Test set (*n *= 35)	Total (*n *= 116)
**Patient characteristics**			
Male (%)	64%	54%	
Age range	16-83	21-82	
Median age	53	56	
**Diagnosis**			
RL	16	7	23
cHL	12	7	19
NHL	53	21	74
DLBCL	8	11	19
FL	25	10	35
Other NHL*	20	0	20

The table summarises the overall number of biopsies for each subtype examined and also the corresponding numbers divided into training and test sets for analysis.

*Includes cases of Burkitt-like lymphoma, MALT lymphoma, mantle cell lymphoma, marginal zone lymphoma, small lymphocytic lymphoma/chronic lymphocytic leukaemia, anaplastic large cell lymphoma, extranodal NK/T-cell lymphoma, and other T-cell lymphomas.

The ability of GEP to ascertain the correct diagnosis of each biopsy was assessed via diagonal linear discriminant analysis (DLDA) with classification error rates in the training set determined by leave one out cross-validation (LOOCV). The ratio of between sum of squares to within sum of squares (*bss/wss*) criteria was used for feature selection performed within each CV fold. As a measure of discriminative power in two-class classification, the selective use of features ranked high in *bss/wss *enriches for potential biomarkers of interest. The top ranked *bss/wss *genes ranging from 10-500 (increments of 10) were assessed within the classification development to identify the number of genes required to obtain a minimal (optimal) cross-validation error rate (see Additional file 1). The classification power of the determined optimal set of genes was then tested on the independent test set sample. Firstly, the results for each classification built from training datasets are expressed in terms of a classification accuracy rate (%), which represents the similarity between the pathological clinical diagnosis and the microarray diagnosis [14]. The accuracy rate of training datasets was determined by subtracting the LOOCV-error rate (%) from 100%. A separate dataset was then used to obtain independent error and accuracy rates. A DLDA classification rule was constructed from the complete training set data using the optimal number of genes estimated via the LOOCV stage of the analysis. This classification rule was then used to classify the independent data. The independent test dataset accuracy rate denotes the percentage of samples in the test dataset that have been correctly diagnosed using the molecular signatures identified from classification of the training dataset.

To examine the feasibility of GEP to classify RL versus lymphoma and subtypes of lymphoma, the expression data was analysed according to three different approaches:

1. A global (all data) multi-class strategy was performed to classify the four main classes examined in this study (RL, cHL, FL and DLBCL) in a single step.

2. A series of independent local (selected data) pair-wise (binary-class) comparisons of the four main classes examined was made. Comparisons included lymphoma versus RL, cHL versus NHL (inclusive of 3 and 17 cases respectively of rare T- and B cell lymphoma subtypes), and lastly, FL versus DLBCL, the two most prevalent forms of NHL in Caucasian populations [10]. These will be refered to as the 'local binary comparisons'

3. A number of 'global binary comparisons' were performed by pair-wise comparisons of samples from an individual subtype versus the remaining data.

For all classification strategies, both LOOCV and independent test set accuracy rates were determined as mentioned above.

### Heat maps

Heat maps of the set of genes (classifiers) yielding the optimal LOOCV accuracy rates were produced. Clustering was performed for both samples and genes utilising hierarchical clustering with Euclidean distance as the dissimilarity function with complete linkage agglomeration. Dendrograms are displayed on the appropriate axis of the heat map.

## Results

### Microarray analysis of lymph node biopsies

Microarrays quantifying gene expression in lymph node biopsies (*n *= 142) suspected of lymphoma were assessed for quality using the arrayQuality package. Arrays with a CV QC score of >1 were identified as sub-optimal in quality and subsequently removed from analysis to result in a final total of 116 arrays analysed. A summary of the diagnosis of each of these biopsies and number of each subtype examined is shown in Table 1.

**Figure 1:**
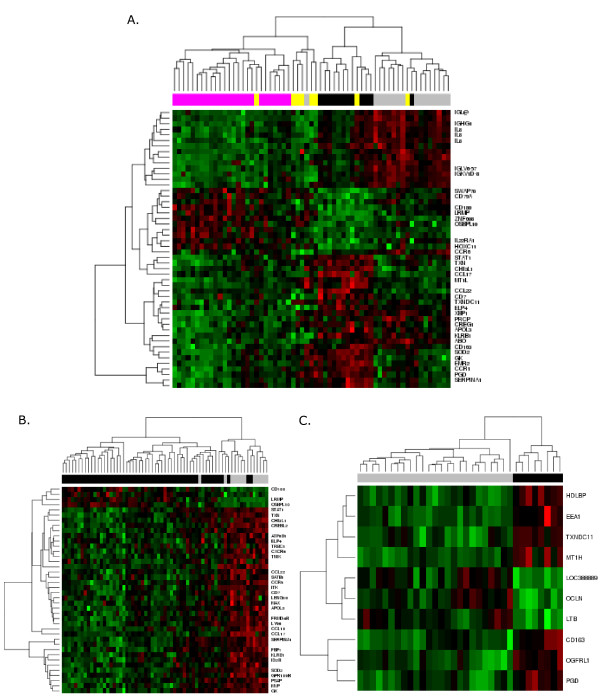
**Hierarchical clustering of lymph node samples comparing distinct subtypes of lymphoma**. Heatmaps depict A. Multi-class analysis for cases of RL (grey, n = 16), cHL (black, n = 12), DLBCL (yellow, n = 8) and FL (magenta, n = 25); B. cHL (grey, n = 12) versus NHL (black, n = 53) and C. FL (grey, n = 25) versus DLBCL (black, n = 8). The columns represent the samples and rows represent the solicited genes. Each cell within the grid is indicative of the gene expression level for an individual sample with colour used to depict intensity on a graduating red (high) to green (low) scale. See Additional files 2 and 3 for the lists of top 10 and 20 classifier genes distinguishing HL from NHL and FL from DLBCL.

A diagnostic profile for the global multi-class approach consisting of 50 genes was developed, and demonstrated an optimal LOOCV accuracy rate of 83.6% for the diagnosis of biopsies as RL, HL, FL or DLBCL (Table 2). Most cases from the diagnostic classes examined in this study clustered distinctly together by hierarchical clustering, with the exception of DLBCL (Figure 1A). An accuracy rate of only 68.6% was achieved when the same profile of genes were tested on an independent test set (Table 2).

**Table 2:** The accuracy rates resulting from GEP classification of lymph node biopsies into selected subtypes.

Comparison	Subtypes	Optimal number of probes	Training set accuracy rate (%)	Test set accuracy rate* (%)
**Global multi-class**	RL v cHL v FL v DLBCL	50	83.6	68.6

**Local binary-class**	RL v Lymphoma	130	87.7	80.0
	cHL v NHL	40	89.2	82.1
	FL v DLBCL	10	84.8	76.1

**Global binary-class**	cHL v remaining cases	30	91.4	82.8
	FL v remaining cases	60	82.7	88.5
	DLBCL v remaining cases	490	87.7	82.8

* The reported independent test set accuracy rates indicate the proportion of correctly classified cases from the total number assessed in the test datasets.

Using local binary classification (lymphoma versus RL, cHL versus NHL, and FL v DLBCL groups; Table 2) LOOCV accuracy rates at each independent comparison ranged from 84.8-89.2%. This high rate of accuracy was reflected in hierarchical clustering analysis, which showed only 5 samples (1 HL and 4 NHL) clustered incorrectly in the NHL versus HL comparison (Figure 1B), and a clear-cut separation between FL and DLBCL samples achieved using only 10 unique classifying genes (Figure 1C). Independent test set accuracy rates for these comparisons were all greater than 76% (Table 2). See Additional files 2 and 3 for the lists of top 10 and 20 classifier genes distinguishing HL from NHL and FL from DLBCL.

Given the higher independent test set accuracy rates when only two defined types of diagnoses (classes) were considered in each analysis, we next investigated if we can diagnose a specific disease type compared to the remainder of all case types examined in this study. The gene classifiers, identified from this type of comparison are likely more specific to the disease type itself, as opposed to signature genes identified purely from a comparison of two subtypes. Samples were classified by comparing each subtype of lymphoma or RL to all remaining cases at the global level and accuracy rates for this type of binary classification determined. Based on varying optimal number of genes, LOOCV accuracy rates of training sets were all above 82%, with test set accuracy rates of 88.5%, 82.8%, 82.8% and 80.0% for FL, cHL, DLBCL and RL respectively.

### Identified gene classifiers of RL, cHL, DLBCL and FL

Strong classifiers of reactive node tissue included the lower expression of a cohort of immune-response related genes compared to tissue diagnosed with lymphoma (Table 3). Reduced expression in reactive node tissue was also observed for genes such as TATA box binding protein (TBP)-associated factor 140 kDa (*TAF3*) and Lim domain binding 2 (*LDB2*). Molecular classifiers identified for cases of cHL include the expression of a selection of chemokine ligands, and the transcription factor *STAT1 *(Table 4). The gene LIM domain only-2 (*LMO2*) was highly differentially expressed in FL compared to the remainder of cases examined (Table 5) whilst high expression of cyclin-dependent kinase inhibitor 3 (*CDKN3*) transcripts was associated with DLBCL (Table 6). A total of 31 genes within any of the four lists of genes identified from binary-class analyses are common to the 38 annotatable genes (of 50 probes) identified from our multi-class analysis. See Additional file 4 for the complete list of classifiers (genes) for all classifications. The clustering of these identified classifiers is not influenced by clinical covariates such as the age or gender (Additional file 5).

**Table 3:** Top 20 annotated classifier genes of RL samples.

Accession number	Gene name	Symbol	Fold change
AF126749	ATXN8 opposite strand (non-protein coding)	*ATXN8OS*	1.65
AL117661	TAF3 RNA polymerase II, TATA box binding protein (TBP)-associated factor, 140 kDa	*TAF3*	0.71
NM_006107	LUC7-like 3 (S. cerevisiae)	*LUC7L3*	0.68
AK025953	Myosin light chain kinase	*MYLK*	0.55
NM_004367	Chemokine (C-C motif) receptor 6	*CCR6*	0.50
X64983	Olfactory receptor, family 10, subfamily D, member 3 pseudogene	*OR10D3P*	0.48
AK024040	Hypothetical LOC148413	*LOC148413*	0.48
AF085877	Hypothetical protein LOC254100	*LOC254100*	0.47
NM_001290	LIM domain binding 2	*LDB2*	0.46
J02639	Serpin peptidase inhibitor, clade A (alpha-1 antiproteinase, antitrypsin), member 5	*SERPINA5*	0.43
NM_002989	Chemokine (C-C motif) ligand 21	*CCL21*	0.42
X87888	Immunoglobulin lambda locus	*IGL@*	0.37
AF026932	Immunoglobulin lambda locus	*IGL@*	0.37
AJ270695	Basic helix-loop-helix family, member e41	*BHLHE41*	0.32
X87890	Immunoglobulin lambda locus	*IGL@*	0.31
U50342	Immunoglobulin kappa constant	*IGKC*	0.29
AF035799	Immunoglobulin kappa variable 3-20	*IGKV3-20*	0.28
AF035787	Immunoglobulin heavy variable 3-48	*IGHV3-48*	0.28
AF035035	Immunoglobulin kappa variable 1D-8	*IGKV1D-8*	0.27
X57772	Immunoglobulin lambda variable 6-57	*IGLV6-57*	0.25

Genes are ranked from high to low fold change (differential expression in reactive versus remainder of samples).

**Table 4:** The top 20 annotated classifier genes of cHL.

Accession number	Gene name	Symbol	Fold change
NM_006152	Lymphoid-restricted membrane protein	*LRMP*	2.83
NM_005582	CD180 molecule	*CD180*	1.65
NM_002382	MYC associated factor X	*MAX*	0.65
NM_006564	Chemokine (C-X-C motif) receptor 6	*CXCR6*	0.57
NM_017458	Major vault protein	*MVP*	0.55
NM_015364	Lymphocyte antigen 96	*LY96*	0.52
D17028	Prosaposin	*PSAP*	0.51
NM_000167	Glycerol kinase	*GK*	0.51
NM_018664	Basic leucine zipper transcription factor, ATF-like 3	*BATF3*	0.48
NM_006137	CD7 molecule	*CD7*	0.45
NM_006018	G protein-coupled receptor 109B	*GPR109B*	0.43
NM_000579	Chemokine (C-C motif) receptor 5	*CCR5*	0.40
NM_003329	Thioredoxin	*TXN*	0.36
M36693	Superoxide dismutase 2, mitochondrial	*SOD2*	0.34
NM_007315	Signal transducer and activator of transcription 1, 91 kDa	*STAT1*	0.34
M26123	Serpin peptidase inhibitor, clade A (alpha-1 antiproteinase, antitrypsin), member 1	*SERPINA1*	0.33
NM_002258	Killer cell lectin-like receptor subfamily B, member 1	*KLRB1*	0.32
NM_002990	Chemokine (C-C motif) ligand 22	*CCL22*	0.29
NM_001276	Chitinase 3-like 1 (cartilage glycoprotein-39)	*CHI3L1*	0.24
NM_002987	Chemokine (C-C motif) ligand 17	*CCL17*	0.18

Genes are ranked from high to low fold change (differential expression in cHL versus remainder of samples).

**Table 5:** The top 20 annotated classifier genes of FL.

Accession number	Gene name	Symbol	Fold change
NM_004244	CD163 molecule	*CD163*	3.20
X57772	Immunoglobulin lambda variable 6-57	*IGLV6-57*	3.03
NM_005502	ATP-binding cassette, sub-family A (ABC1), member 1	*ABCA1*	1.86
NM_005080	X-box binding protein 1	*XBP1*	1.86
NM_020397	Calcium/calmodulin-dependent protein kinase ID	*CAMK1D*	1.83
NM_000153	Galactosylceramidase	*GALC*	1.43
AF298812	Ectodysplasin A2 receptor	*EDA2R*	0.72
NM_013340	Protocadherin beta 1	*PCDHB1*	0.72
AB046800	Leucine rich repeat containing 4C	*LRRC4C*	0.70
NM_016524	Synaptotagmin XVII	*SYT17*	0.70
NM_005582	CD180 molecule	*CD180*	0.69
NM_014212	Homeobox C11	*HOXC11*	0.64
NM_014146	Linker for activation of T cells family, member 2	*LAT2*	0.63
AK001057	Hypothetical LOC114130	*MGC16384*	0.63
NM_006822	RAB40B, member RAS oncogene family	*RAB40B*	0.59
NM_002753	Mitogen-activated protein kinase 10	*MAPK10*	0.55
NM_000319	Peroxisomal biogenesis factor 5	*PEX5*	0.49
AB033107	Zinc finger protein 608	*ZNF608*	0.46
NM_000869	5-hydroxytryptamine (serotonin) receptor 3A	*HTR3A*	0.38
NM_005574	LIM domain only 2 (rhombotin-like 1)	*LMO2*	0.37

Genes are ranked from high to low fold change (differential expression in FL versus remainder of samples).

**Table 6:** The top 20 annotated classifier genes of DLBCL.

Accession number	Gene name	Symbol	Fold change
NM_000439	Proprotein convertase subtilisin/kexin type 1	*RBM16*	2.03
AF111846	Transcribed locus	*CCNB1*	1.54
NM_000492	Cystic fibrosis transmembrane conductance regulator (ATP-binding cassette sub-family C, member 7)	*TFRC*	0.66
NM_017421	Coenzyme Q3 homolog, methyltransferase (S. cerevisiae)	*SLC25A4*	0.65
AL137452	Protein arginine methyltransferase 10 (putative)	*PGD*	0.63
NM_016138	Coenzyme Q7 homolog, ubiquinone (yeast)	*CDKN3*	0.63
NM_014726	TBK1 binding protein 1	*CCT8*	0.63
AL049705	Mitochondrial ribosomal protein S14	*FKBP4*	0.61
NM_018320	Ring finger protein 121	*BZW2*	0.61
NM_003566	Early endosome antigen 1	*CCT3*	0.60
NM_006231	Polymerase (DNA directed), epsilon	*GMPS*	0.56
NM_015902	Ubiquitin protein ligase E3 component n-recognin 5	*MSI1*	0.56
NM_002626	Phosphofructokinase, liver	*MMRN1*	0.55
NM_001634	Adenosylmethionine decarboxylase 1	*SLMAP*	0.55
NM_006476	ATP synthase, H+ transporting, mitochondrial F0 complex, subunit G	*NDUFV1*	0.53
NM_007159	Sarcolemma associated protein	*SBNO1*	0.53
NM_006330	Lysophospholipase I	*ESPL1*	0.51
NM_003384	Vaccinia related kinase 1	*MYO19*	0.49
NM_006585	Chaperonin containing TCP1, subunit 8 (theta)	*CYC1*	0.47
NM_001151	Solute carrier family 25 (mitochondrial carrier; adenine nucleotide translocator), member 4	*ANAPC5*	0.47

Genes are ranked from high to low fold change (differential expression in DLBCL versus remainder of samples).

## Discussion

In the present study, we used GEP microarrays to analyse 116 lymph node biopsies to assess the feasibility of this technology as a diagnostic tool in a clinical setting. This study is preceded by a significant body of research on GEP of lymphoma that has focused on understanding the pathogenesis of individual subtypes of lymphoma and refining the diagnosis and prognosis of these subtypes. However, our aim was to examine the practical question of whether GEP could be used to classify lymph node samples into the major subtypes of lymphoma and also to distinguish them from reactive lymph nodes.

The ability of GEP to diagnose biopsies of reactive, cHL, DLBCL and FL origin was examined with three strategies: global multi-class classification; local binary-class and global binary-class classification. The global multi-class approach classified each sample into one of the four diagnostic types with limited accuracy, which is known to decrease when more than two classes are considered simultaneously in linear classification algorithms [15]. Our binary comparisons, which compared a particular diagnostic type with either another type (local) or with the remainder of all cases (global), resulted in high (>80%) accuracy rates for independent test sets, except when comparing FL to DLBCL (76.1%), the subtype that was most frequently misclassified. This limitation of GEP in classifying DLBCL may be related to the high degree of heterogeneity of the disease itself. Distinct molecular forms of DLBCL have been identified in other GEP studies [4,16,17], although this does not readily explain the misclassified cases of this study, which included both GCB and non-GCB DLBCL as judged by the Hans algorithm for immunohistochemistry. As the partial involvement of a tissue biopsy by lymphoma cannot be excluded, sampling error may also contribute to classification error rates. In regards to the comparison of RL with lymphoma, the two RL samples misclassified were both reactive hyperplasia. It should be noted that our reactive nodes were unselected and as such not all of which would necessarily have been B-cell predominant reactions. Therefore the random sampling of reactive nodes, which have different compartments, may contribute to sampling error. The accuracy to distinguish benign from malignant may be improved by increasing the number of cases used to build the classification, especially since there is an imbalance in the number of reactive biopsies (23) compared to the number of cancerous cases (93).

Application of our findings to clinical practice would require a much larger scale study to not only verify our identified genetic signature of particular types but also to assess the profile of uncommon lymphoma subtypes. We nonetheless feel that this work represents an important step in testing the principle of using GEP, based on simple and inexpensive arrays, as a diagnostic ancillary test for lymph node biopsy. We found that our laboratory practices were easily adapted to allow routine allocation of a portion of biopsy specimen for microarray as routine tests such as flow cytometry and cytogenetics, for diagnosis of lymphoma, also require fresh specimen (not formalin fixed). The development of new techniques such as quantitative nuclease protection assays on formalin-fixed, paraffin-embedded tissue blocks would overcome any difficulty in obtaining fresh tissue for microarray gene expression profiling and make GEP much more widely available even in small biopsies [18].

The 18% technical exclusion rate of samples arrayed in this study hampers the diagnostic utility of microarray. However, increased familiarity with the assay will reduce the exclusion rate, and in laboratories with a limited caseload, referral to a centralised service may be preferable. Given the substantial improvement of microarray technology since the initiation of this study, the use of newer genome-wide microarray platforms such as Illumina bead arrays would also improve the utility of this technology and contribute to reducing the technical exclusion rate seen in this study. Incorporation of microRNA array data [19] may also be appropriate, especially given the reported stability of microRNA expression [20].

In our study, 13 of the 40 classifier genes identified from a specific (local) comparison of cHL with NHL were also strong classifiers when cHL was globally compared to both NHL and reactive samples. This indicates that our classification strategy encompasses unique gene sets that can classify across more than two types of pathological conditions. Although some gene classifiers identified in our study were common to other reported GEP studies, the absence of some previously identified key classifiers may be due to variable probe make-up across different microarray platforms or resulting from differences in the type of diagnostic classes used in our classification compared to most published GEP studies [21]. Our distinct global binary comparisons would have likely identified gene signatures that represent the particular diagnostic type in question as it was compared to a mixture of lymphoma subtypes and non-cancerous samples.

The fact that high expression of *CD7*, *CCL17 *and *STAT1 *has been reported to be associated with cHL supports the reliability of our microarray data presented in this study [22-24]. As Hodgkin and Reed-Sternberg cells only account for on average 1% of the mixed cell types present in HL infiltrates, it is likely that the expression of some of the HL classifiers are derived from the stromal cell population. This should not influence the applicability of lymph node GEP to the diagnosis of HL given that this stromal reaction is likely to be similar across different HL samples and that their gene expression profiles have been reported to predict the outcome of HL [25]. Similarly for FL, our detected reduced expression of *CD163*, a macrophage marker, may reflect a low number of macrophages present in the node microenvironment in many cases of FL. The importance of this information is not diminished as increased reactive macrophages in a rare subset of FL have been reported to be associated with poorer survival [26]. *LMO2*, another strong molecular classifier identified for FL, has been reported to be expressed in approximately 50% of FL [27]. However, it is better known as a key gene expressed in GCB cell type of DLBCL [4] and as a strong predictor of superior outcome in DLBCL [28]. Given the importance of *LMO2 *expression in DLBCL, its absence in our list of top 20 classifying genes of DLBCL may be due to the fact that only 5 cases examined (26%) are of GCB cell origin by immunohistochemistry. Instead, we have identified the gene cyclin D kinase inhibitor 3 (*CDKN3*), a known marker of the ABC-like DLBCL [29], to be expressed higher in our DLBCL samples compared to the other diagnostic types examined in this study.

The lower expression of several immunoglobulin genes in reactive node tissue may reflect the differences in the cellular makeup of the microenvironment of normal lymph node tissue compared to those diseased with lymphoma. Consistent with the phenotype of non-cancerous tissue, we detected reduced expressions of a potentially cancerous gene *TAF3*, a negative regulator of the tumour suppressor p53 [30].

## Conclusions

This proof of principle study has shown that microarray as a single platform assay can achieve reasonable diagnostic accuracy with the ability to differentiate lymphoma from non-cancerous reactive lymphadenopathy, and also classify three common subtypes of lymphoma. Molecular classifiers identified to be characteristic of each subtype examined could be combined onto a cost effective custom 'mini' microarray to screen lymph node biopsies for expression profiles to assist the diagnosis of four common outcomes: reactive, cHL, FL or DLBCL in a relatively quick and inexpensive manner. Indeed the use of GEP as a diagnostic and prediction tool for other diseases is currently commercially available. One example includes the CupPrint and MammaPrint array service provided by Agendia for patients with breast cancer. The increased sensitivity in RNA extraction techniques has made GEP much more widely available even for paraffin sections, small fine needle aspiration and core biopsies, which are routinely used in a pathology lab. The application of GEP may streamline current diagnostic tests by allowing more selective use of costly and time consuming ancillary tests such as immunohistochemistry, immunophenotyping by flow cytometry, cytogenetics, FISH and PCR in every case of lymphoma. Its use may provide an objective diagnostic test that could be standardised across pathology laboratories.

## Competing interests

The authors declare that they have no competing interests.

## Authors' contributions

THL, TJM, and ML carried out the microarray experiments and interpretation of results. AC and JYHY carried out the computational and statistical analysis. AB participated in the histopathology review and interpretation of data. JT provided expert histopathology review and interpretation of data. DDFM participated in study design, guidance in analysis and interpretation of data. All authors have contributed to and approved the manuscript.

## Pre-publication history

The pre-publication history for this paper can be accessed here:

http://www.biomedcentral.com/1755-8794/4/27/prepub

## Supplementary Material

Additional file 1 **Determination of optimal error rates**. a pdf file describing how the optimal error rates for each classification was obtained. Click here for file

Additional file 2 **Classifier genes that distinguish cHL from NHL**. a pdf file containing one table Click here for file

Additional file 3 **Classifier genes that distinguish FL from DLBCL**. a pdf file containing one table. Click here for file

Additional file 4 **Complete list of all classifier genes**. an excel file containing 7 spreadsheets. Click here for file

Additional file 5 **Dendrogram analysis of clinical covariates**. A pdf file showing that the classifiers are not biased towards or against clinical covariates using dendrogram analysis. Click here for file
