# Supplementary material for: Discriminating lymphomas and reactive lymphadenopathy in lymph node biopsies by gene expression profiling
Source: BMC Med Genomics. 2011 Mar 31;4:27. doi: 10.1186/1755-8794-4-27 (PMC3080274; doi:10.1186/1755-8794-4-27)
Supplement: Additional file 1 — Determination of optimal error rates. a pdf file describing how the optimal error rates for each classification was obtained. [file 1755-8794-4-27-S1.PDF]

**Optimal error rates in classification:**

Consensus rates of training datasets was determined by subtracting the LOOCV-error rate (%) from 100%. The influence of gene set numbers on the consensus rates determined for each comparison are highlighted in plots graphing consensus rates versus the number of top ranking genes used for analysis. As an example, the result from the FL versus remainder comparison is shown in Figure A1. For this particular comparison, a gradual decrease in the overall consensus rate was observed when the number of genes included in analysis increased. As the overall consensus rate was highest for 60 genes (probes), this gene set list was chosen for the optimal consensus rate. Assessment of both overall and split consensus rates over an increasing number of ranked genes enables the determination of the optimal gene set which results in the lowest error rate overall.

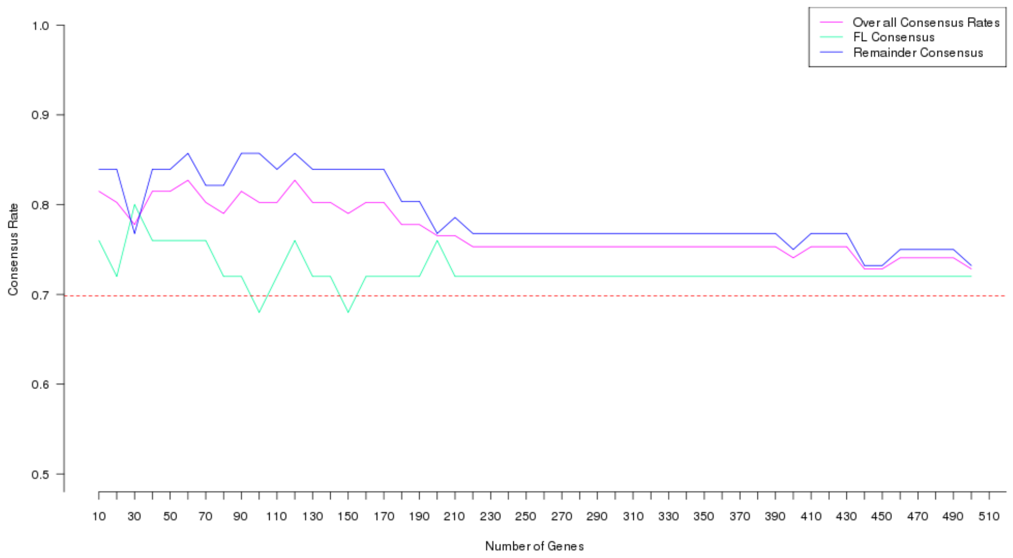


**Figure A1:** Consensus rates of classification of lymphoma and reactive samples across the top 500 ranked genes. Three consensus rates are plotted, denoting the overall and the two split consensus rates, which are defined as the proportion of agreement resulting for each subtype.
