# Supplementary material for: Discriminating lymphomas and reactive lymphadenopathy in lymph node biopsies by gene expression profiling
Source: BMC Med Genomics. 2011 Mar 31;4:27. doi: 10.1186/1755-8794-4-27 (PMC3080274; doi:10.1186/1755-8794-4-27)
Supplement: Additional file 2 — Classifier genes that distinguish cHL from NHL. a pdf file containing one table [file 1755-8794-4-27-S2.PDF]

Table A2. Top 20 annotated classifier genes that distinguish cHL from NHL.

Accession number	Gene name	Symbol	Fold change
NM_002987	Chemokine (C-C motif) ligand 17	*CCL17*	5.24
NM_002988	Chemokine (C-C motif) ligand 18 (pulmonary and activation-regulated)	*CCL18*	4.48
NM_001276	Chitinase 3-like 1 (cartilage glycoprotein-39)	*CHI3L1*	3.78
NM_002258	Killer cell lectin-like receptor subfamily B, member 1	*KLRB1*	3.64
NM_002990	Chemokine (C-C motif) ligand 22	*CCL22*	3.36
M26123	Serpin peptidase inhibitor, clade A (alpha-1 antiproteinase, antitrypsin), member 1	*SERPINA1*	3.16
NM_007315	Signal transducer and activator of transcription 1, 91kDa	*STAT1*	2.99
M36693	Superoxide dismutase 2, mitochondrial	*SOD2*	2.97
NM_003329	Thioredoxin	*TXN*	2.85
NM_002971	SATB homeobox 1	*SATB1*	2.60
NM_006137	CD7 molecule	*CD7*	2.32
NM_001310	CAMP responsive element binding protein-like 2	*CREBL2*	2.00
NM_000167	Glycerol kinase	*GK*	1.90
AF172264	TRAF2 and NCK interacting kinase	*TNIK*	1.87
NM_006564	Chemokine (C-X-C motif) receptor 6	*CXCR6*	1.71
Z77831	Interleukin 23, alpha subunit p19	*IL23A*	1.64
NM_019040	Elongation protein 4 homolog (S. cerevisiae)	*ELP4*	1.48
NM_002382	MYC associated factor X	*MAX*	1.57
NM_006152	Lymphoid-restricted membrane protein	*LRMP*	0.34
NM_017784	Oxysterol binding protein-like 10	*OSBPL10*	0.31

Genes are ranked from high to low fold change (differential expression in cHL versus NHL samples).
