# Supplementary material for: Discriminating lymphomas and reactive lymphadenopathy in lymph node biopsies by gene expression profiling
Source: BMC Med Genomics. 2011 Mar 31;4:27. doi: 10.1186/1755-8794-4-27 (PMC3080274; doi:10.1186/1755-8794-4-27)
Supplement: Additional file 3 — Classifier genes that distinguish FL from DLBCL. a pdf file containing one table. [file 1755-8794-4-27-S3.PDF]

**Table A1**. The 10 annotated classifier genes that distinguish FL from DLBCL.

| Accession number | Gene name | Symbol | Fold change |
|---|---|---|---|
| NM_004244 | CD163 molecule | *CD163* | 4.33 |
| X64177 | Metallothionein 1H | *MT1H* | 2.83 |
| AK024732 | Opioid growth factor receptor-like 1 | *OGFRL1* | 2.28 |
| NM_002631 | Phosphogluconate dehydrogenase | *PGD* | 2.11 |
| NM_015914 | Thioredoxin domain containing 11 | *TXNDC11* | 1.86 |
| NM_003566 | Early endosome antigen 1 | *EEA1* | 1.79 |
| NM_005336 | High density lipoprotein binding protein | *HDLBP* | 1.57 |
| Z70716 | Occludin | *OCLN* | 0.60 |
| AK026502 | Hypothetical LOC388889 | *LOC388889* | 0.59 |
| NM_002341 | Lymphotoxin beta (TNF superfamily, member 3) | *LTB* | 0.50 |

Genes are ranked from high to low fold change (differential expression in FL versus DLBCL samples).
