# Supplementary material for: Discriminating lymphomas and reactive lymphadenopathy in lymph node biopsies by gene expression profiling
Source: BMC Med Genomics. 2011 Mar 31;4:27. doi: 10.1186/1755-8794-4-27 (PMC3080274; doi:10.1186/1755-8794-4-27)
Supplement: Additional file 5 — Dendrogram analysis of clinical covariates. A pdf file showing that the classifiers are not biased towards or against clinical covariates using dendrogram analysis. [file 1755-8794-4-27-S5.PDF]

Heatmap analysis of clinical and experimental covariates:

To show that the identified classifiers are not biased towards or against any clinical or experimental covariates, we firstly clustered the relevant samples using the classifiers identified in all comparisons made in this study. We then colour coded the samples below each dendrogram according to gender, age, hospital source, date sample was arrayed, diagnosis and subtype/grading. In all dendrograms, samples were found to be randomly spread according to the different covariate features thus indicating that the identified classifiers are not biased towards/against any of these factors. The dendrograms for the FL versus DLBCL and the cHL versus remainder comparison are shown as examples in the dendrograms below (Figure A2).

<u>FL versus DLBCL (10 classifiers)</u>

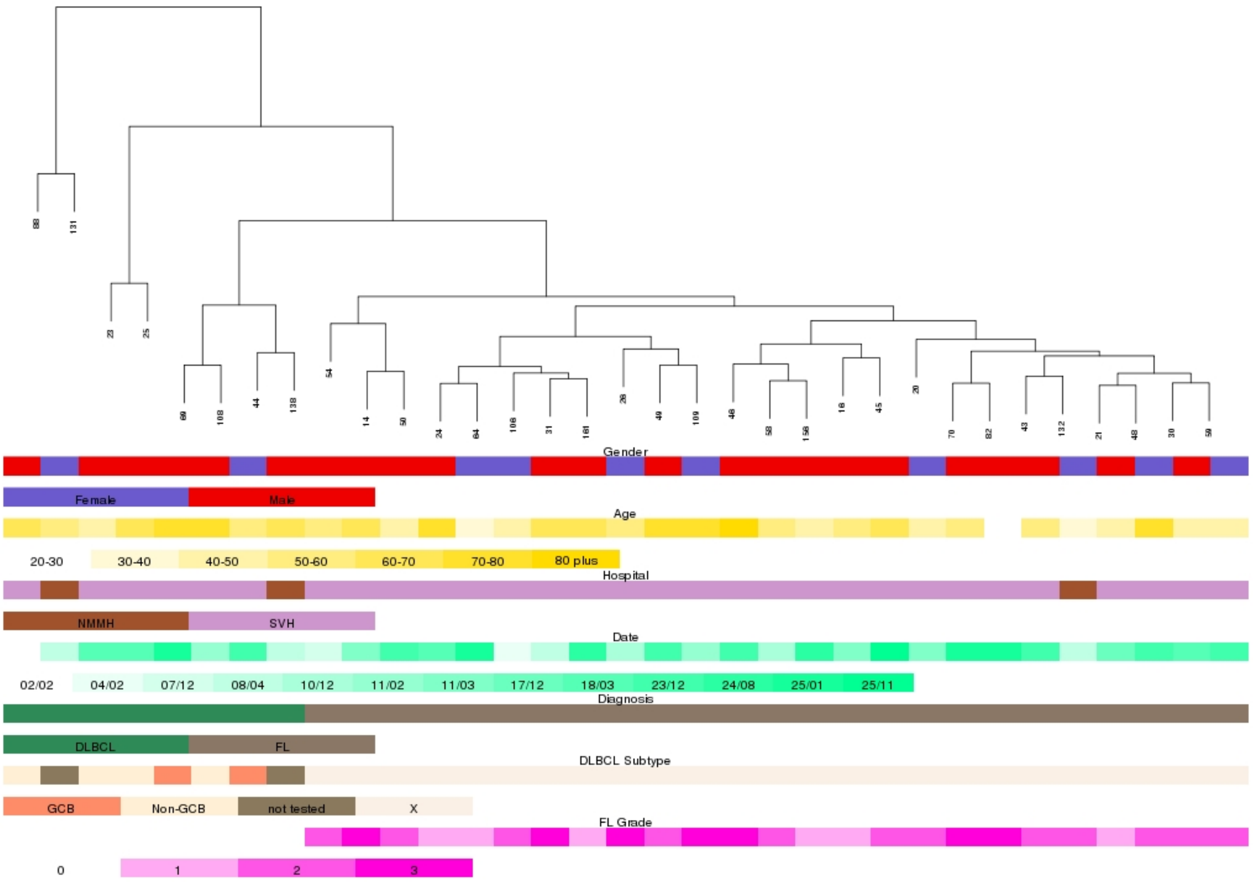

cHL versus remainder (40 classifiers)

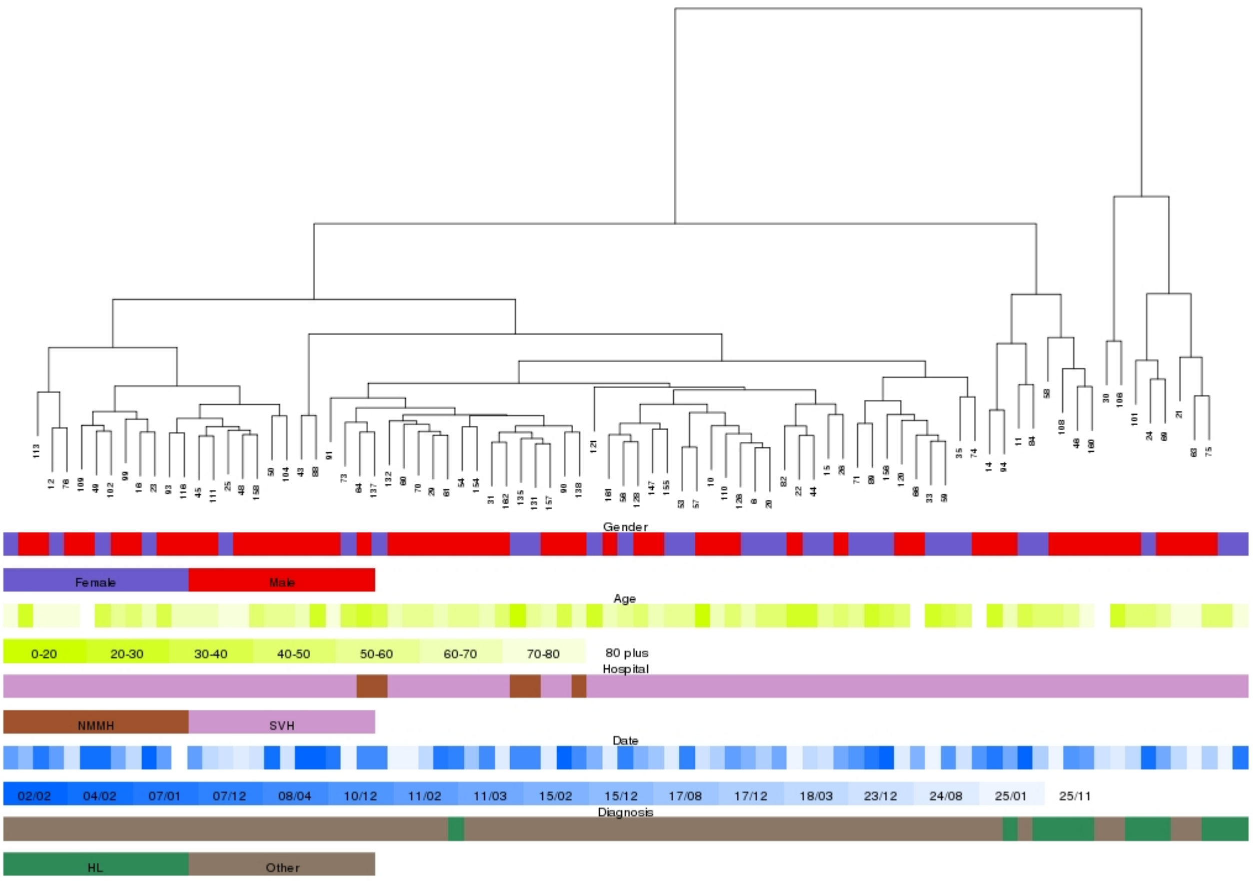

Figure A2: Analysis of clinical covariates below dendrograms. Samples from the FL versus DLBCL or cHL versus remainder comparison were clustered using the classifiers identified from these comparisons. Depicted below each dendrogram is the random clustering of samples colour coded according to gender, age, hospital (where tissue was biopsied) and date sample was arrayed, diagnosis, FL grading or DLBCL subtype.
